# Fixed Drug Eruptions in Malagasy Children: Clinical Observations and Characteristics

**DOI:** 10.1155/crdm/6580695

**Published:** 2026-01-31

**Authors:** Fenohasina Rakotonandrasana, Fandresena Arilala Sendrasoa, Onivola Raharolahy, Stevy Desana, Voahanginirina Nathalie Ralimalala, Tsiory Iarintsoa Razafimaharo, Moril Sata, Malalaniaina Andrianarison, Lala Soavina Ramarozatovo, Fahafahantsoa Rapelanoro Rabenja, Irina Mamisoa Ranaivo

**Affiliations:** ^1^ Department of Dermatology, Place Kabary Hospital, Antsiranana, Madagascar; ^2^ Department of Dermatology, Joseph Raseta Befelatanana Hospital, Antananarivo, Madagascar; ^3^ Department of Dermatology, Morafeno Hospital, Toamasina, Madagascar

**Keywords:** drug eruption, fixed drug eruption, pediatrics, pigmented dermatosis

## Abstract

Fixed drug eruption is a drug‐induced hypersensitivity reaction characterized by recurrent erythematous‐pigmented lesions at the same site after each exposure to the causative drug. The molecules most frequently implicated are sulfonamides, nonsteroidal anti‐inflammatory drugs, anticonvulsants, and paracetamol. The first case involved a four‐year‐old boy with sickle cell disease who had presented with a recurrent hyperpigmented macule on the lip for 1 year. The second case involved a three‐year‐old girl with multiple pigmented, pruritic macules. The third case involved a ten‐year‐old boy presenting with pigmented plaques and flaccid bullae on his arm and left thigh. In all three cases, fixed drug eruption (including one bullous form) was diagnosed based on the patient history and recurrence. Management consisted of permanent withdrawing of the offending drug and providing symptomatic treatment with antihistamines and topical corticosteroids. There were favorable outcomes, but persistent residual pigmentation remained. These three cases illustrate the typical clinical presentation of fixed drug eruption in children in Madagascar. Recurrence of the same lesion at the same site is pathognomonic and requires discontinuation of the offending drug and reporting to pharmacovigilance.

## 1. Introduction

Fixed drug eruption (FDE) is a drug‐induced hypersensitivity reaction, characterized by the occurrence of erythematous‐pigmented plaques, sometimes bullous, which recur at the same site after exposure to the same sensitizing drug [1]. The most frequently implicated medications are sulfonamides, nonsteroidal anti‐inflammatory drugs, anticonvulsants, and paracetamol [1, 2]. It most often affects young to middle‐aged adults, aged 35 and 60 years. In children, it accounts for 14%–22% of drug‐induced skin eruptions [3].

We report three cases of FDE in children seen at the department of Dermatology of Place Kabary Hospital, Antsiranana, Madagascar.

## 2. Case Reports

### 2.1. Case 1

**FIGURE 1:**
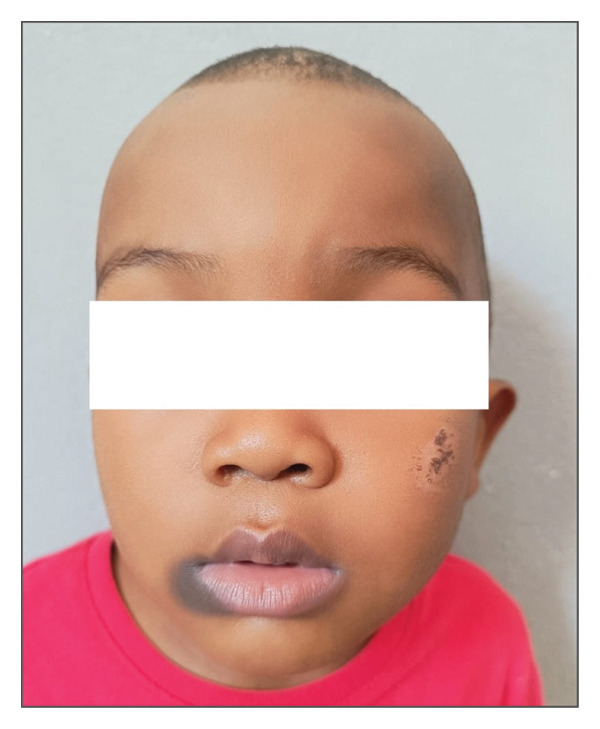
Well‐defined hyperpigmented macule on the right labial commissure. *Source:* Department of dermatology, Place Kabary Hospital, Antsiranana.

A 4‐year‐old boy with sickle cell disease presented with a well‐defined, asymptomatic hyperpigmented, finely scaly, macule located at the right labial commissure, evolving for 1 year (Figure 1). According to his mother, he had received paracetamol 250 mg every 6 h for 2 days to treat fever 1 week prior to consultation. A rapid recurrence of the lesion, strictly at the same site, was observed 24 h after drug intake. The evolution was marked by persistence of postinflammatory pigmentation. Causality assessment using the Naranjo scores yielded a score of 9, indicating a definite relationship between paracetamol and the lesion.

### 2.2. Case 2

**Figure 2:**
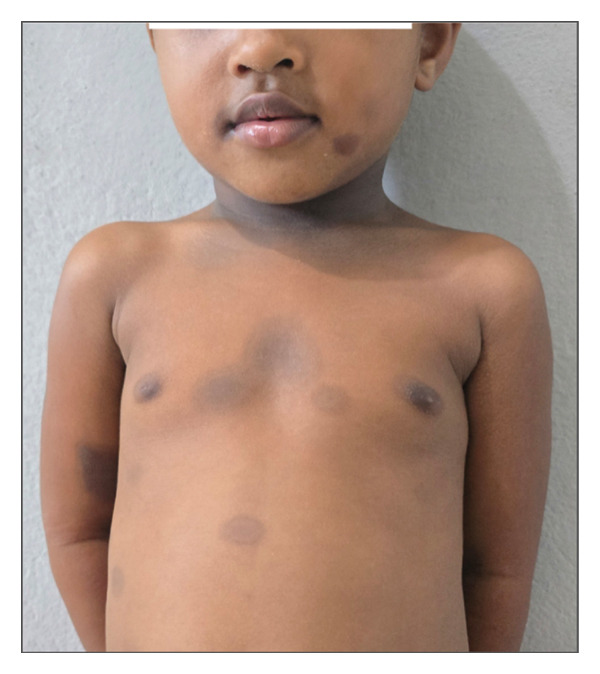
Well‐defined hyperpigmented macules on the face, neck, thoracoabdominal region, and anterior surface of the right arm. *Source:* Department of dermatology, Place Kabary Hospital, Antsiranana.

A 3‐year‐old girl, with no notable medical history, was brought in for macules and sometimes pigmented plaques, well‐circumscribed and limited by pigmented ridges. The lesions are pruritic and located at the right orbital region, right labial commissure, trunk, neck, and upper limbs (Figure 2). These lesions had been recurrent for 1 year. The most recent episode occurred 8 days earlier, following administration of sulfamethoxazole–trimethoprim prescribed for a febrile infectious episode. Assessment of drug causality using the Naranjo scores resulted in a score of 10, confirming a definite link between sulfamethoxazole–trimethoprim and the lesions.

### 2.3. Case 3

**Figure 3:**
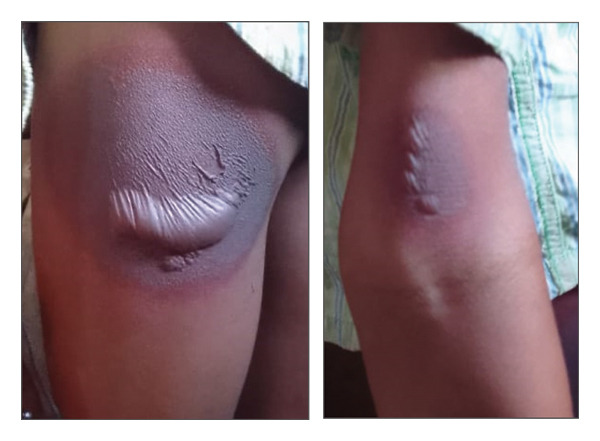
Pigmented plaques topped with flaccid bullae on the anterior surface of the left arm and the posteroexternal aspect of the left thigh. *Source:* Department of dermatology, Place Kabary Hospital, Antsiranana.

A 10‐year‐old boy, with no significant medical history, consulted for recurrent pruritic skin lesions evolving for six months. Dermatological examination revealed well‐demarcated pigmented plaques with erythematous borders, surmounted by flaccid, clear‐content bullae, located on the anterior surface of the left arm, and the posteroexternal aspect of the left thigh (Figure 3). The lesions appeared approximately 12 h after ingestion of paracetamol. The bullae resolved spontaneously, leaving residual brownish pigmentation. Assessment of drug causality using the Naranjo score resulted in a score of 8, suggesting a probable link between paracetamol and the lesions.

In all three cases, the diagnoses of FDE and bullous FDE were supported by the clear temporal relationship between drug intake and lesion onset, the recurrent pattern of the eruptions, and Naranjo scores ranging from 8 to 10. No drug provocation tests were performed due to strong clinical suspicion. From a therapeutic perspective, immediate and definitive withdrawal of the offending drugs was the first measure.

Symptomatic treatment was initiated, consisting of oral antihistamines for pruritus and topical corticosteroids. The outcome was favorable in all cases, with progressive resolution of the lesions.

## 3. Discussion

FDE represents a well‐defined entity among drug‐induced hypersensitivity reactions. The pathophysiological mechanism involves memory T lymphocytes located in the epidermis and superficial dermis, which specifically react to the drug or its metabolites upon each exposure [1, 4].

Lesions typically present as erythematous‐pigmented macules, sometimes associated with bullae or vesicles, evolving into prolonged residual hyperpigmentation. The latency period for onset ranges from a few hours to several days. The topography is variable, but sites such as the mouth, extremities, genital organs, or photo‐exposed areas are frequently affected [5, 6].

Drug causality was assessed using the Naranjo scores, a tool widely employed in case reports to standardize the evaluation of causal relationships. In our series, the scores ranged from probable to definite, strengthening the implication of the suspected drug in the occurrence of the lesions [7].

The diagnosis of FDE relies primarily on the patient’s history, particularly the temporal relationship between drug intake and lesion onset, as well as the topographic recurrence of lesions upon re‐exposure. Controlled drug reintroduction may be considered in a specialized setting, but only when identification of the causative drug is uncertain and therapeutic alternatives are limited. In doubtful cases, a skin biopsy may be performed. Patch testing can be proposed but has variable sensitivity and may be poorly tolerated in young children [1, 6, 8].

Management relies primarily on the definitive withdrawal of the offending drug. Antihistamines or topical corticosteroids may be used to relieve symptoms in the acute phase. Patient and family education, along with notification to the pharmacovigilance center, are essential to prevent future accidental exposures [1, 9].

## 4. Conclusion

FDE is an avoidable but underdiagnosed cutaneous drug reaction. Our cases highlight the importance of raising awareness among families and healthcare providers about this diagnosis, ensuring close follow‐up, and strengthening pharmacovigilance systems in order to prevent accidental re‐exposures and better document the local epidemiology of this condition.

**Nomenclature**
FDE: Fixed drug eruption

## Author Contributions

Conception and design: Fenohasina Rakotonandrasana, Stevy Desana, Voahanginirina Nathalie Ralimalala, and Tsiory Iarintsoa Razafimaharo.

Review and editing: Fenohasina Rakotonandrasan, Moril Sata, Onivola Raharolahy, Malalaniaina Andrianarison, and Fandresena Arilala Sendrasoa.

Final approval of the version to be published: Irina Mamisoa Ranaivo, Lala Soavina Ramarozatovo, and Fahafahantsoa Rapelanoro Rabenja.

## Funding

The authors received no specific funding for this work.

## Consent

No written consent has been obtained from the patients as there are no patient identifiable data included in this case report.

## Conflicts of Interest

The authors declare no conflicts of interest.

## Data Availability

The data that support the findings of this study are available on request from the corresponding author.
